# Diagnosis and treatment of schizotypal personality disorder: evidence from a systematic review

**DOI:** 10.1038/s41537-018-0062-8

**Published:** 2018-10-03

**Authors:** Sophie K. Kirchner, Astrid Roeh, Jana Nolden, Alkomiet Hasan

**Affiliations:** Department of Psychiatry and Psychotherapy, Ludwig-Maximilians-University, Munich, Germany

## Abstract

The main objective of this review was to evaluate studies on the diagnosis, treatment, and course of schizotypal personality disorder and to provide a clinical guidance on the basis of that evaluation. A systematic search in the PubMed/MEDLINE databases was conducted. Two independent reviewers extracted and assessed the quality of the data. A total of 54 studies were eligible for inclusion: 18 were on diagnostic instruments; 22, on pharmacological treatment; 3, on psychotherapy; and 13, on the longitudinal course of the disease. We identified several suitable and reliable questionnaires for screening (PDQ-4+ and SPQ) and diagnosing (SIDP, SIDP-R, and SCID-II) schizotypal personality disorder. Second-generation antipsychotics (mainly risperidone) were the most often studied drug class and were described as beneficial. Studies on the longitudinal course described a moderate remission rate and possible conversion rates to other schizophrenia spectrum disorders. Because of the heterogeneity of the studies and the small sample sizes, it is not yet possible to make evidence-based recommendations for treatment. This is a systematic evaluation of diagnostic instruments and treatment studies in schizotypal personality disorder. We conclude that there is currently only limited evidence on which to base treatment decisions in this disorder. Larger interventional trials are needed to provide the data for evidence-based recommendations.

## Introduction

Schizotypy is a heterogenous syndrome that is expressed across multiple dimensions, including cognitive-perceptual, disorganized, and interpersonal symptoms^1^ or according to the symptomology of schizophrenia, positive, negative, and disorganized factors.^2–4^ Raine^1^ described two types of schizotypy: (1) neurodevelopmental schizotypy with relatively stable traits and significant brain and neurocognitive impairments that predispose to schizophrenia, and (2) pseudoschizotypy, a pronounced psychosocial entity with more symptom fluctuation that is unrelated to schizophrenia. Schizotypy, as a broader collection of both clinical and nonclinical traits, is assessed by psychometric inventories such as the Wisconsin Scales of Schizotypy.^3^ The assessments for schizotypal traits are mainly used to define a high-risk group and its proneness to psychosis.^5^ Mason described 16 different schizotypy scales that were based on clinical concepts or definitions and six scales for psychometric/personality measures of schizotypy.^5^

According to the theoretical models of Meehl,^6,7^ Lenzenweger,^8^ Chapman,^9^ and Kwapil,^10^ schizotypy is a premorbid condition. The term schizotypy refers to both people with schizotypal personality disorder (STPD) and healthy individuals in the general population with certain personality traits and a latent liability for psychosis.^1^ Consequently, research has been performed in both clinical patients and healthy schizotypal individuals. Several authors argue for a dimensional approach and a continuum between schizotypal traits and schizophrenia spectrum disorder^1,11,12^; support for their argument is provided by genetic and linkage studies showing a considerable overlap between genetic association profiles in schizotypy and schizophrenia.^13,14^

Since the introduction of the diagnosis of STPD in Diagnostic and Statistical Manual of Mental Disorders, Third Edition (DSM-III) in 1980, the diagnosis and treatment of STPD have remained difficult because of the lack of evidence-based algorithms. The original STPD item set was derived from the criteria of borderline schizophrenia seen in the relatives of schizophrenia patients. The differentiation between schizotypal traits and STPD is clinically important and reflects the degree of impairment in occupational and interpersonal functioning and the severity of symptom presentation.^14^ This review focuses on the diagnosis of and therapeutic approaches in patients with a disease severity that fulfills the criteria of STPD not only as a premorbid condition or risk state but also as a separate diagnostic entity.

For years, the International Classification of Diseases (ICD) from the World Health Organization (WHO) and other classification instruments, e.g., the DSM from the American Psychiatric Association (APA), have differed in their classification of STPD (referred to as *schizotypal disorder* in the ICD): In ICD-9 and -10, it is listed under schizophrenia spectrum disorders, whereas in DSM-III to -5 it is classified as a personality disorder. In the forthcoming ICD-11, it will remain in the block of schizophrenia spectrum disorders.^15^ In DSM-5, a diagnosis of STPD is defined by the following symptom categories: (1) general impairments in personality and self-functioning (identity and self-direction) and in interpersonal functioning (empathy, intimacy); (2) STPD-specific pathological personality traits, described as psychoticism, eccentricity, cognitive and perceptual dysregulation, and unusual beliefs and experiences, (3) detachment characterized by restricted affectivity and withdrawal, and (4) negative affectivity characterized by suspiciousness. In ICD-10,^16^ and very likely in ICD-11, however, schizotypal disorder (F21) is characterized by eccentric behavior and changes in thinking and affect similar to those in schizophrenia; the evolution and course of the disease resemble those of a personality disorder (PD).

The prevalence of STPD has been described as ranging from 0.6% in a Norwegian sample to 4.6% in an American sample.^17^ Men (4.2%) are more often affected than women (3.7%).^17^ Common differential diagnoses are other PDs such as the borderline personality disorder (BPD), attention-deficit disorder (inattentive type), social anxiety disorder, autism-spectrum disorder, and dysthymia.^18^ Comorbidities can complicate the disease course and treatment responses, and several studies focus on interventions for patients with comorbid obsessive-compulsive disorder (OCD)^19^ and BPD. Schizotypy occurs more often in relatives of patients with schizophrenia or a Cluster A PD. Twin studies showed highly stable genetic factors and rather transient environmental factors for an increased risk for the schizotypal syndrome,^20^ and genetic risk variants for schizophrenia could also be linked to STPD.^21–23^ The conversion rates from STPD to schizophrenia spectrum disorders vary between 20 and >40%, depending on the follow-up interval.^1,24^ Imaging studies detected numerous group-level differences in the size of specific brain regions in individuals with STPD or schizotypy in comparison with healthy participants, patients with schizophrenia, and patients with other PDs.^18^

Despite these research efforts, evidence-based recommendations are still lacking for the diagnosis and treatment of STPD. National and international treatment guidelines for schizophrenia spectrum disorders (e.g., from the APA, the National Institute for Health and Care Excellence and World Federation of Societies of Biological Psychiatry (WFSBP)) do not discuss this topic, and specific guidelines for personality disorders pay only little attention to STPD.^25^ Rosell et al.^18^ recently published a non-systematic literature review on the epidemiology, functional impairment, heritability and genetics, cognitive impairments, social-affective disturbances, and neurobiology of STPD. This detailed review provides a deeper insight into the pathophysiology of and experimental research on STPD and includes information on imaging and genetic and psychological testing.^18^ Nevertheless, it remains unclear which diagnostic tools, medication, or psychotherapy are recommended. Therefore, the main objective of this systematic review, which was based in principle on the recommendation of the Preferred Reporting Items for Systematic Reviews and Meta-Analyses group,^26^ was to evaluate the literature on the diagnosis and treatment of patients seeking help for STPD. In addition, it offers some information on the longitudinal course of STPD and conversion rates to other schizophrenia spectrum disorders.

## Results

### Articles on diagnostic instruments

#### Study characteristics

When examining the articles on diagnostic instruments, we focused on studies that tested disease criteria^27–31^ and clinical diagnostic questionnaires for STPD.^32–39^ One study did not specifically evaluate a diagnostic questionnaire but compared a factor analysis model with established questionnaires.^40^

#### Individual outcomes

Five studies aimed at evaluating the variables of the diagnostic criteria, the shifts in diagnosis from DSM-III to -5 or ICD-8 to -10 and their effect on diagnostic sensitivity, specificity, and diagnostic overlap,^27,28,30,31,41^ as indicated in Table 1. The reliability of the DSM-III criteria had an adequate mean kappa of 0.71.^27^ In DSM-III, the diagnoses of BPD and STPD interacted on the symptom level.^27^ In DSM-III-R, the threshold for an STPD diagnosis was raised, and the number of STPD diagnoses consequently decreased by 40%. This offered a sharper discrimination between STPD and related PDs, such as BPD and schizoid PD.^30^ In the evolution of the ICD system, the threshold for meeting symptom criteria was also crucial for differential diagnosis: Whereas in ICD-8/-9 less patients would have been diagnosed with STPD, in ICD-10 the threshold was lowered and more patients were diagnosed with STPD. In the ICD system, the severity of the disease may mark the discrimination between STPD and schizophrenia, which would favor a dimensional diagnosis concept.^31^ Most of the studies evaluated the inter-rater reliability (which ranged from 0.62 to 0.91) and test–retest reliabilities (which ranged from 0.64 to 0.84) of the diagnostic instruments; reliabilities were adequate (see Table 1). Among the diagnostic instruments, three were identified as being suitable for diagnosis because they had adequate reliabilities and validities for the respective diagnostic criteria: the Structured Interview for DSM-III Personality Disorder (SIDP),^33^ the Semi-structured Interview for DSM-III-R Personality Disorders (SIDP-R),^35^ and the Structured Clinical Interview for DSM-IV (SCID-II)^37^ (see Table 1). The self-report instruments Schizotypal Personality Questionnaire (SPQ) and Schizotypal Personality Questionnaire—Brief (SPQ-B) were designed as screening instruments for STPD. The SPQ-B shows adequate internal consistency (coefficient kappa = 0.87),^42^ and the SPQ shows strong correlations between patients’ responses and SCID-II–rated symptoms.^38,43^ The Personality Diagnostic Questionnaire-4+ (PDQ-4+) is also a self-report instrument for diagnosing PD. It has more false-positive results than the SCID-II and is therefore only useful as a screening tool for PDs but not as a diagnostic tool.^44^ The Minnesota Multiphasic Personality Inventory (MMPI) could not differentiate between individuals with STPD and schizophrenia.^45^ However, a distinct profile (MMPI 2-7-8) showed an enrichment of diagnoses of Cluster A PDs when compared with SIDP-IV interviews. None of the instruments was designed to evaluate disease severity. Three studies compared diagnostic interviews with factor analysis models.^35,40,44^ According to Battaglia et al.^35^, the three factors cognitive-perceptual, interpersonal, and oddness best describe the diagnosis of STPD; Fossati et al.^37^ made the same statement, but these two studies had overlapping patient cohorts. Sanislow et al.^40^ developed a four-factor model and argued that it was significantly better than the established unitary “generic” model.^40^ Please see Table 1 for details.

**Table 1:** Qualitative synthesis of diagnostic instruments for schizotypal personality disorder

Reference	Study population	Questionnaire	Outcome^a^
Perry et al.^34^	*n*_total_ = 19 psychiatric patients	Schedule for Schizotypal Personalities (SSP), DSM-III symptom criteria	The SSP is a structured interview developed by Baron for identifying relatives of chronic schizophrenic patients. Here it is tested if the SSP is also suitable for clinical patients. The reliability coefficients of the statement scores for the SSP range from *I*_R_ = 0.68 to 0.99. Using the score from the SSP, the inter-rater reliability of making a diagnosis of SPD was *I*_R_ = 1.0. Using the author’s individual ratings for making clinical diagnosis of SPD, the *I*_R_ was 0.72. The reliability for the single items was found reliable and valid with the exception of odd speech. The retest reliability for the SSP of the SPD diagnosis was *I*_R_ = 0.84. The retest reliability for the authors’ consensus diagnoses was not obtained. The authors’ clinical consensus was used as the criterion for determining the validity of the diagnosis: The validity of the SSP depends on the cutoff for symptom criteria. At the cutoff of 2 criteria, the diagnostic sensitivity is 100% and the specificity is 9%. At the cutoff of 4 criteria, the diagnostic sensitivity decreased to 13% and the specificity rose to 100%. For the evaluation of the diagnostic validity of the DSM-III criteria, the authors’ consensus judgement was compared with the diagnosis based on DSM-III criteria. The sensitivity per item ranges between 25 and 88% (except for the criterion recurrent illusion) and the specificity is for all items >80%. The authors recommend combining the criteria in four conceptual categories: (a) self-report of cognitive-perceptual disturbances, (b) observable disorder of thought/communication, (c) deficits in drive/affect, and (d) interpersonal difficulties
Stangl et al.^33^	*n*_total_ = 131 psychiatric patients, *n*_PD_ = 67, *n*_SPD_ = 12	Structured Interview for DSM-III Personality Disorder (SIDP)	The SIDP is a semi-structured interview. It is designed to assess the DSM-III-R PD diagnoses. It was developed to improve Axis II diagnostic reliability. 63 patients were retested and rated by two raters. The kappa coefficient for SPD was 0.62. The authors argue that the validity cannot be measured since the SIDP is based on the DSM-III criteria. The DSM-III criteria have not been tested for validity and another gold standard is missing
Jacobsberg et al.^27^	*n*_total_ = 64 psychiatric patients, *n*_SPD_ = 35	DSM-III criteria, Global Assessment Scale (GAS), Schedule for Interviewing Borderline Patients	The reliability for identifying schizotypal symptoms by DSM-III criteria showed a mean kappa = 0.71. Patients with schizotypal personality disorder showed a lower GAS score than patients with other personality disorders. In this clinical sample, DSM-III symptoms of schizotypal and borderline personality disorder interact. The following symptoms were significantly related with the diagnosis of schizotypal personality disorder compared to other personality disorders: inadequate rapport (*φ* = 0.61), odd communication (*φ* = 0.54), chronic boredom (*φ* = 0.48), depersonalisation (*φ* = 0.45), social isolation (*φ* = 0.45), illusions (*φ* = 0.44), ideas of reference (*φ* = 0.38), identity disturbance (*φ* = 0.33), undue mood elevation upon praise (*φ* = 0.33),paranoid ideation (*φ* = 0.32), magical thinking (*φ* = 0.32), compulsive sociability (*φ* = 0.28), and delusions (*φ* = 0.27). Patients with schizotypal diagnosis vary in their symptoms depending on whether these patients have been drawn from clinical or familial samples
Widiger et al.^28^	*n*_total_ = 84 psychiatric patients	DSM-III criteria	The internal consistency and descriptive validity of the borderline and schizotypal symptoms were analyzed by calculating (a) their sensitivity, specificity, positive predictive power, and negative predictive power, and (b) their correlation with the diagnoses’ cutoff points and total number of symptoms. Most of the symptoms were successful as inclusion tests but not as exclusion tests. The social-interpersonal symptoms were less efficient in differentiating schizotypal from borderline patients than the perceptual-cognitive symptoms. The schizotypal symptom combinations had consistently high positive predictive power (PPP) rates. None was <0.81, with the exception of social anxiety-hypersensitivity and social isolation, which obtained a PPP of only 0.73. The means of the PPP and specificity rates for SPD were 0.92 (SD = 0 .07) and 0.95 (SD = 0.06) and the mean negative predictive power (NPP) and sensitivity rates for SPD were 0.53 (SD = 0.04) and 0.39 (SD = 0.11)
McGlashan^29,41^	*n*_total_ = 109 psychiatric patients (SPD and/or BPD), *n*_SPDonly_ = 10	DSM-III symptom criteria	The most characteristic symptoms for schizotypal personality disorder are odd communication, suspiciousness/paranoid ideation, and social isolation. The least discriminating symptoms are illusions/depersonalisation/derealisation. Whereas depression as a symptom does not discriminate between SPD and BPD, transient psychoses and brief paranoid experiences fit better as SPD criteria
Vaglum et al.^30^	*n*_total_ = 97 psychiatric patients, *n*_SPD_ = 21 (by DSM-III rating), *n*_SPD_ = 13 (by DSM-III-R rating)	DSM-III and DSM-III-R criteria	The inter-rater reliability is for DSM-III and DSM-III-R equally good (kappa value for DSM-III diagnosis is 0.64 and for DSM-III-R of 0.67). When diagnosed by DSM-III-R criteria, the number of SPD diagnosed was reduced by 40% compared to DSM-III criteria. Of the schizotypal patients, who lost their diagnosis in DSM-III-R (*n* = 8), 4 got a new diagnosis of schizoid personality disorder and 4 maintained their comorbid borderline personality disorder
Raine^34^	Sample (1): *n*_1_ = 302 population-based subjects used for the development of the test	Preliminary Schizotypal Personality Questionnaire (SPQ), Present State Evaluation (PSE), Scale for the Assessment of Negative Symptoms (SANS), Structured Clinical Interview for DSM-III-R Personality Disorders (SCID-II), Schedule for Affective Disorders and Schizophrenia (SADS)	The SPQ is a self-report measure for all nine feature categories of the schizotypal personality disorder as defined by DSM-III-R. Items for the SPQ were generated from the PSE, SANS, SCID-II, and SADS. Additionally, some items of the STA scale, the Schizotypy scale, the Perceptual Aberration scale, and the Magical Ideation scale were included. The SPQ was found to have high internal reliability (0.91), retest reliability (0.82), discriminant validity (0.63), and criterion validity (0.68). 55% of the subjects scoring in the top 10% of the SPQ had a clinical diagnosis of schizotypal personality disorder. The authors conclude that the SPQ might be a good tool for screening for schizotypal personality disorder
	Sample (2): *n*_2_ = 195 population-based subjects used to test the replicability of results generated in the first sample	Schizotypal Personality Questionnaire (SPQ), Structured Clinical Interview for DSM-III-R Personality Disorders (SCID-II)	
Battaglia et al.^35^	*n*_1_ = 538 nonpsychotic psychiatric patients, *n*_2_ = 225 non-patient controls	Italian version of Semistructured Interview for DSM-III-R Personality Disorders (SIDP-R)	The SIDP-R is a structured interview that covers the full DSM-III-R range of PDs. The mean kappa value, chance corrected for SIDP-R-generated Axis II diagnoses, was 0.83; the mean kappa value for SIDP-R diagnosis of SPD was 0.89. Three factors encompass the diagnosis best: cognitive-perceptual, interpersonal, and oddness
Merrit et al.^36^	*n*_total_ = 4000 students, *n*_2-7-8_ = 38	Minnesota Multiphasic Personality Inventory (MMPI), Structured Interview for DSM-IV Personality Disorder (SIDP-IV)	Participants with a distinct profile on MMPI (2-7-8) received higher scores of personality disorder diagnoses than controls. 85% of the 2-7-8 group received a Cluster A diagnosis. 50% of participants with a 2-7-8 profile received the diagnosis of schizotypal personality disorder according to DSM-IV criteria measured by SIDP-IV
Fossati et al.^37^	*n*_total_ = 564 psychiatric patients, *n*_SPD_ = 30 (by SCID-II rating)	Structured Clinical Interview for DSM-IV (SCID II)	The SCID-II is a structured interview for DSM-IV criteria for all personality disorders. The inter-rater reliability was adequate for all SPD criteria (median kappa = 0.846). SPD is best explained by four latent classes, but only the latent class I (“no close friends,” odd thinking,” “suspiciousness,” and “inappropriate affect”) is associated with SPD diagnosed by DSM-IV. The most discriminating DSM-IV SPD criterion was “odd thinking”
Sanislow et al.^40^	*n*_total_ = 668 psychiatric patients	Structured Clinical Interview for DSM-IV Axis-I Disorders–Patient Version (SCID-I/P), Diagnostic Interview for DSM-IV Personality Disorders (DIPD-IV)	Confirmatory factor analysis was used to test the DSM-IV construct of the borderline, schizotypal, avoidant, and obsessive-compulsive disorder. A model based on the three DSM-IV Axis II clusters was also tested. Both models were tested against a unitary ‘generic’ model constructed from four criteria sets combined. Median kappa coefficients ranged from 0.58 to 1.0 for all Axis II disorders. Based on factor analysis, a one-factor, three-factor and a four-factor-model were established. The four-factor model provided an analysis acceptable fit to the data (χ2 (489) = 1756.8; CFI = 0.83, NFI = 0.78 and RMSEA = 0.062). Overall, results from this study support the division of DSM-IV personality disorders into at least four disorders over a unidimensional model of personality disorder (based on the numerous studies documenting high co-occurrence among DSM-IV personality disorders), and over a three-factor model of personality disorder (based on the DSM-IV Axis II clusters).
Axelrod et al.^42^	*n* = 237 psychiatric patients, *n*_PD_ = 34	Schizotypal Personality Questionnaire Brief (SPQ-B)	The SBQ-B is a self-report instrument. It is modeled after the DSM-III-R schizotypal personality disorder diagnostic criteria (largely unchanged in DSM-IV). It was developed to study schizotypal personality patterns and to screen for schizotypal personality disorder in the general community. It includes cognitive-perceptual deficits, interpersonal deficits, and disorganization. The SBQ-B shows adequate internal consistency (coefficient alpha = 0.87). The Interpersonal and Cognitive-Perceptual subscales demonstrates convergent and discriminant relationships with other measures of interpersonal impairment and cognitive abnormalities. The Personality Disorder group had significantly greater Interpersonal Deficit scores than the Conduct Disorder and Substance Use Disorders groups (*p* < 0.05)
Matsui et al.^45^	*n*_total_ = 321 psychiatric patients and healthy controls, *n*_SPD_ = 18, *n*_SZ_ = 41, *n*_students_ = 262	Minnesota Multiphasic Personality Inventory (MMPI)	Discriminant function analysis of the MMPI profiles revealed significant variance among the three groups. The overall rate of correct classification of the subjects into schizophrenia, SPD, or university students was 90.3%. Yet, there is a considerable overlap between schizophrenia and SPD
Dickey et al.^38^	*n*_SPD_ = 104	Structured Clinical Interview for DSM-IV (SCID II), Schizotypal Personality Questionnaire (SPQ)	Inter-rater reliability for diagnosis of SPD based on SCID interviews of 25 subjects and 3 raters showed a kappa of 0.89. The most common SPD criteria met was impairment due to experiencing unusual perceptions, followed by suspiciousness/paranoid ideation and odd beliefs/magical thinking. There were strong correlations observed between SPD subjects’ self-report on the SPQ and SCID interview for the Cognitive Perceptual (*r* = 0.616, *N* = 41, *p* < 0.0005) and the Interpersonal (*r* = 0.406, *N* = 41, *p* = 0.008) factors but not for the Disorganized factor (*r* = 0.003, *N* = 41, *p* = 0.99)
Fossati et al.^44^	Study 1: *n*_total_ = 721 psychiatric patients, *n*_SPD_ = 31 (by SCID-II rating), *n*_SPD_ = 166 (by PDQ-4+ rating) Study 2: *n*_2_ = 537 psychiatric patients, *n*_SPD_ = 16	Personality Diagnostic Questionnaire-4+ (PDQ-4+), Structured Clinical Interview for DSM-IV (SCID-II) DSM-III-R Personality Disorders-Revised (SIDP-R), Diagnostic Interview Schedule-Revised (DIS-R)	The SCID II is a structured interview for DSM-IV criteria for all personality disorders. The PDQ-4+ is a forced choice, self-report, 99-item questionnaire designed to measure the DSM-IV PDs. In these samples, schizophrenia-spectrum disorders, mental retardation, or dementia were excluded. The inter-rater reliability for diagnosis of SPD based on SCID II interviews of 25 subjects and 3 raters was kappa = 9.1. PDQ-4+ seems to predict false-positive results. The factor model shows schizophrenia-related schizophrenia. PDQ-4+ scales do not identify subjects with definite personality disorder diagnoses. Reliability and predictive power improved when SPD was assessed by semi-structured, direct interviews that used all nine diagnostic criteria instead of through self-assessments of some dimensions of schizotypy. Considering SCID-II data, Raine’s three-factor model of SPD features (Raine et al., 1994) was the best fitting model among those considered in study 1. Oddness was the factor that most sharply discriminated the SPD latent taxon from the complement in the clinical subjects of this study. The DIS-R is a diagnostic interview for Axis I disorders. 16 subjects (3.0%) in study 2 were diagnosed with DSM-III-R SPD; this was similar to the number of SPD subjects in study 1, *χ*^2^ (1) = 1.15, *p* > 0.25. In line with a previous confirmatory factor analysis, the cognitive-perceptual, interpersonal, and oddness three-factor model of SPD features was the best fitting model in this sample. The sample of Study 2 has already been explored by Battaglia et al.^35^.
	Study 3: *n*_3_ = 225 non-patient controls, *n*_SPD_ = 2	DSM-III-R Personality Disorders-Revised (SIDP-R)	SIDP-R is a semi-structured, 160- item interview divided into 16 thematic sections; it is designed to assess the DSM-III-R PDs.
Handest et al.^31^	*n*_total_ = 151 psychiatric patients, *n*_SPD_ = 50	ICD-10 criteria, ICD-8/9 criteria, Operational Criteria for Psychotic Illness (OPCRIT), Bonn Scale for Assessment of Basic Symptoms (BSABS), Positive and Negative Syndrome Scale (PANSS), DSM-IV Global Assessment of Functioning Scale (GAF-F)	If the ICD-10 criteria threshold was lowered or elevated, the number of individuals diagnosed with schizotypal disorder would vastly differ (from 14 to 86 patients in this cohort). Factor analysis resulted in four factors: interpersonal/negative, disorganized, perceptual/positive, and paranoid symptoms. Compared to the schizophrenia subgroup, schizotypal patients scored higher on depression (OR = 6.65, 95% CI 2.52–17.60) and sleep disturbances (OR = 4.91, 95% CI 1.65–14.57) and lower on suspiciousness (OR = 0.28, 95% CI 0.12–0.63), loss of role functioning (OR = 0.17,95% CI 0.06–0.44), and odd behavior (OR = 0.42,95% 0.19–0.95). If diagnosed by the ICD-8/9 criteria, only 37 patients (out of 50) would have been diagnosed with schizotypal disorder. The other 12 patients would have been diagnosed with schizophrenic disorder by the ICD-8/9 criteria. The severity of psychosis marks the distinction of schizophrenia from schizotypy. The study shows the arbitrary nature of the four criteria needed for the ICD–10 schizotypy diagnosis. Schizophrenia and schizotypy are similar on most psychopathological dimensions
Ryder et al.^43^	*n*_total_ = 203 psychiatric patients, *n*_PD_ = 70	Structured Clinical Interview for DSM-IV Personality Disorders (SCID-II), Revised NEO Personality Inventory (NEO PI-R), Structured Clinical Interview for DSM-IV, Axis I Disorders (SCID-I), Global Assessment of Functioning (GAF)	The SCID-II PD items were evaluated for all personality disorders with respect to coherence, discrimination, relations with personality traits, and functional impairments. For SPD, the SCID-II showed a convergent validity of 0.64, divergent validity of 0.45, a relation to personality dimension of 0.18, and a relation to impairment of 0.36. Schizotypal PD items met three of the four criteria but showed no relation to Five Factor Model of Personality dimensions, suggesting that it may be a candidate for reassignment to Axis I
Morey et al.^39^	*n*_total_ = 337 patients, *n*_SPD_ = 32	Personality Inventory for DSM-5 (PID-5), Personality Diagnostic Questionnaire-4 (PDQ-4) for DSM-IV	The PID-5 is a self-report questionnaire based on DSM-5 criteria. The PDQ-4+ is a forced choice, self-report, 99-item questionnaire designed to measure the DSM-IV PDs. The patients were additionally rated by clinicians on a four-point trait rating scale. The trait assignments specified for personality disorders in the DSM-5 alternative model demonstrated substantial correlations with the corresponding DSM‐IV diagnoses, with an average trait–disorder correlation of 0.52. The DSM-5 alternative model features are roughly comparable with DSM-IV criterion-derived diagnoses with the *κ*s (median *κ* = 0.50) obtained when comparing DSM-IV criterion-derived diagnoses with the global DSM-IV clinical diagnoses made by these same clinicians. The diagnostic rules for schizotypal personality disorder include three traits of the psychoticism domain (cognitive and perceptual dysregulation, unusual beliefs and experiences, and eccentricity), which demonstrated correlations between 0.67 and 0.61

^a^The descriptions of outcome are direct citations or extracts from the referred publications

### Articles on interventional drug treatment trials

#### Study characteristics

Most of the studies were prospective double-blind, placebo-controlled trials,^46–56^ and all publications reported single-center results. We also identified one double-blind, treatment-controlled trial;^57^ one single-blind, placebo-controlled trial;^58^ four open-label trials;^59–62^ one retrospective study;^63^ and four case reports.^64–67^ Please see Table 2A, B and Supplementary Table 3 and 4 for further study details and level of evidence (LoE) grading. In total, 16 individual drug treatments were studied. The most frequently studied drug was risperidone,^50,53,54,62^ and the most frequently studied class of drugs were the antipsychotics,^47,48,50,53,54,57–60,62,63,66^ followed by the antidepressants.^46,61,64,65^ Six studies tested other neuroactive drugs.^49,51,52,55,56,67^ In most studies, the main outcome measurement was a general psychiatric symptom scale, such as the Brief Psychiatric Rating Scale,^60,63,67^ the Hopkins Symptom Checklist,^48,61^ and the Psychiatric Assessment Interview.^57^ Three trials used interviews for specific psychotic symptoms, such as the Positive and Negative Symptom Scale (PANSS).^50,53,56^ Some studies focused on patients with comorbidities of OCD or BPD and consequently measured outcome with the Yale-Brown Obsessive Compulsive Scale (YBOCS)^46,48,59,66^ or the Self-Injurious Behavior scale.^47^ One study used a specific tool, the SPQ, to assess the severity of STPD as an additional outcome measurement tool.^50^ Common secondary outcome tools were measurements for global functioning, i.e., the Global Assessment of Symptoms^60^ and Clinical Global Impression,^50^ or for depressive symptoms, i.e., the Hamilton Rating Scale for Depression.^46,50,62^ Six studies had cognitive measures, i.e., working memory,^49,52,55^ context processing,^51^ reaction time,^54^ response inhibition,^54^ or a combination of different cognitive categories,^53^ as a primary or secondary outcome.

**Table 2:** Qualitative synthesis of drug treatment trials

(A) Antipsychotics											
Reference	Study population	Study type	Inclusion criteria	Exclusion criteria	Intervention	Symptoms measured	Mean pretreatment	Mean posttreatment	Symptom change	Outcome^a^	Level of evidence (risks of bias LoE according to SIGN)
Serban et al.^57^	*n*_total_ = 52, *n*_STPDonly_ = 14, *n*_STPD+BPD_ = 16	3-month, double-blind, treatment-controlled trial	Diagnosis of BPD or STPD by DSM-III criteria, disease symptoms >2 years, mild transient psychotic episode before admission	Schizophrenia or affective disorder	Thiothexine hydrocloride (dose 9.4 ± 7.6 mg/day) or haloperidole (3 ± 0.8 mg/day)	Psychiatric Assessment Interview (for *n*_STPDonly_): general symptoms (thiothexine group)			Change in score: mean −0.93	The results indicate that properly defined groups of schizotypal and borderline patients respond well to treatment with tranquilizers, particularly thiothixene. The main areas of symptom control appear to be related to general symptoms, cognitive disturbance, paranoid ideation, anxiety, and depression. There was no significant relationship between diagnosis and outcome of treatment	No randomization. Various outcomes. Monocentric study. LoE: 2−
						General symptoms (haloperidole group)			Change in score: mean −0.08		
						Depression (thiothexine group)			Change in score: mean −0.83		
						Depression (haloperidole group)			Change in score: mean −0.68		
						Paranoia (thiothexine group)			Change in score: mean −1.28		
						Paranoia (haloperidole group)			Change in score: mean −0.75		
						HAMD (for *n*_STPDonly_)					
						(thiothexine group)			Change in score: mean −12.67		
						(haloperidole group)			Change in score: mean −10.40		
Goldberg et al.^47^	*n*_total_ = 50 (BPD and STPD; 24 in treatment group, 26 in placebo group), *n*_STPDonly_ = 13 (6 in treatment group, 7 in placebo group)	12-week double-blind, placebo-controlled trial	Diagnosis of BPD or STPD by DSM-III criteria (rated by Schedule for Interviewing Borderliners SIB), at least one psychotic symptom	Diagnosis of schizophrenia, mania, and melancholia; severe hepatic, renal, or cardiovascular disease; organic brain symptom and/or mental retardation; history of epilepsy or seizures; glaucoma; current alcoholism or drug addiction; severe hypertensive or hypotensive cardiovascular disease; severe metabolic disorders	Low-dose thiothexine hydrocloride (mean dose 8.7 mg/day)	Global Assessment Scale (GAS) (for *n*_total_ = 24)	61.67	72.42	1.16^b^	Significant drug placebo differences were found, regardless of diagnosis, on “illusions,” “ideas of reference,” “psychoticism,” “obsessive-compulsive symptoms,” and “phobic anxiety” but not on “depression”	No randomization. Various outcomes. Monocentric study. LoE: 2−
						HSCL-90 scale: phobic anxiety (for *n*_total_ = 24)	0.5556	0.2222	0.4823^b^	^b^The symptom change is measured as standardized change: (pretreatment score−posttreatment score)/pretreatment standard deviation	
						Phobic anxiety (for *n*_STPD_ = 6)	0.42	0.16	0.26^b^		
						Obsessive compulsive (for *n*_total_ = 24)	1.1429	0.7083	0.504^b^		
						Obsessive compulsive (for *n*_STPD_ = 6)	1.38	0.48	0.6^b^		
						Psychoticism (for *n*_total_ = 24)	1.98	0.77	1.21^b^		
						Psychoticism (for *n*_STPD_ = 6)	1.14	0.36	0.73^b^		
						SIB					
						Illusions (for *n*_total_ = 24)	1.4271	1.2083	0.2715^b^		
						Illusions (for *n*_STPD_ = 6)	1.25	1.16	0.09^b^		
						Ideas of reference (for *n*_total_ = 24)	1.75	1.1806	0.7463^b^		
						ideas of reference (for *n*_STPD_ = 6)	1.72	1.00	0.72^b^		
						Schizotypal symptoms (for *n*_total_ = 24)	15.9062	12.7483	0.7106^b^		
						Psychotic (for *n*_total_ = 24)	9.1493	7.0937	0.6182^b^		
Hymowitz et al.^58^	*n*_STPD_ = 17	2-week single-blind placebo-controlled trial	Diagnosis of BPD by DSM-III criteria (rated by Schedule for Interviewing Borderliners SIB)	Axis I diagnosis of schizophrenia or major affective illness (by DSM-III criteria)	Haloperidole (mean dose 3.6 mg/day)	SIB			Decline in total schizotypal score (*t* = −2.4401, df = 15, *p* < 0.05).	Patients experienced a decline in their total schizotypal score. Among the ten individual schizotypal SIB scales, ideas of reference, odd communication, and social isolation decreased significantly. The total borderline SIB scores, based on the subsequent 11 SIB scales declined, but to a nonsignificant extent (*t* = LoE−1.1861). Thus the statistically significant results reflect mild-to-moderate improvement in approximately half of the patients. Analysis of the drop-outs not only indicated suspiciousness and reluctance about taking any medication but also repeated complaints of side effects, even at the 2 mg level of haloperidole	No adequate concealment (single-blind). No randomization. Various outcomes. Monocentric study. LoE: 2−
Goldberg et al.^48^	*n*_total_ = 50 (BPD and/or STPD)	12-week double-blind placebo-controlled trial	Diagnosis of BPD and/or STPD by DSM-III criteria (rated by Schedule for Interviewing Borderliners SIB), at least one psychotic symptom	Diagnosis of schizophrenia, mania, and melancholia; severe hepatic, renal, or cardiovascular disease; organic brain symptom and/or mental retardation; history of epilepsy or seizures; glaucoma; current alcoholism or drug addiction; severe hypertensive or hypotensive cardiovascular disease; severe metabolic disorders	Thiothexine hydrocloride (mean dose 8.7 mg/day)					Drug-treated patients respond to medication regardless of their MMPI profile. The MMPI scales validity (F), depression (D), psychopathic deviate (Pd), psychasthenia (Pt), paranoia (Pa), and schizophrenia (Sc) are insufficient to predict outcome of drug response. The primary outcome variables of the same cohort measuring the drug–placebo differences have already been published in Goldberg et al. (1986)	Various outcomes. Monocentric study. LoE: 2+
Bogetto et al.^59^	*n*_total_ = 23 patients with non-responsive OCD, *n*_STPD_ = 4	12-week open-label augmentation trial (after 6 month acute treatment)	YBOCS score >16, HAMD score <5 (on the 17-item scale), completion of Structured Clinical Interview for DSM IV Personality Disorders (SCID-II), ill for >1 year, completed an acute treatment phase, non-responder status	Major depressive disorder, dementia, delirium, amnestic or other cognitive disorders; schizophrenia or other psychotic disorders and bipolar disorders; concomitant pharmacotherapy or psychotherapy; general medical condition that would contraindicate the use of fluvoxamine or olanzapine	Fluvoxamine (300 mg/day)+augmentation with olanzapine (5 mg/day)	Mean duration of illness (for *n*_total_)	11.5 ± 9.8			A significant decrease of mean YBOCS score between pretreatment and posttreatment 26.8 ± 3.0 vs. 18.9 ± 5.9 was found at the end point. The rate of responders in the sample was 43.5%. Four of the 10 responders (40%) had a concomitant schizotypal personality disorder. None of the non-responders had a schizotypal personality disorder. A significant difference was found only for the Axis II co-diagnosis (*p* = 0.024). Concomitant schizotypal personality disorder was the only factor significantly associated with response. It appears that augmentation of olanzapine in fluvoxamine-refractory OCD may be effective in a large number of patients, including those with comorbid schizotypal personality disorder	No concealment. No randomization. Various outcomes. Monocentric study. LoE: 3
						Mean YBOCS score (for *n*_total_)	26.8 ± 3.0	18.9 ± 5.9	Mean decrease: 8.0 ± 5.6		
Koenigsberg et al.^50^	*n*_STPD_ = 25	9-week randomized, double-blind placebo-controlled trial	Diagnosis of STPD by DSM-IV criteria rated by the Schedule for Interviewing DSM-IV Personality Disorders (SIDP-IV)	Diagnosis of schizophrenia, schizophrenia-related psychotic disorder, or bipolar disorder; comorbid borderline personality disorder; medical or neurological illness; alcohol or substance abuse within the past 6 months or history of past substance dependence; free of psychotropic medication	Low-dose risperidone (0.25–2 mg/day)	PANSS total			Reduction of 29%	Patients receiving active medication showed a significant decline of symptoms (*p* < 0.05) on the PANSS negative and general symptom scales by week 3 and on the PANSS positive symptom scale by week 7 compared with patients receiving placebo. PANSS scores at baseline are not specifically mentioned and only shown in figures (PANSS total ≈ 60–70, PANSS negative ≈ 15–20, PANSS general ≈ 30–35, PANSS positive ≈ 15). Side effects were well tolerated. There was no group difference in drop-out rates. Conclusion: Low-dose risperidone appears to be effective in reducing symptom severity in STPD patients and is generally well tolerated	Various outcomes. Monocentric study. LoE: 2++
						PANSS negative			Reduction of 27%		
						PANSS general			Reduction of 30%		
						PANSS positive			Reduction of 27%		
						SPQ score	28.2 ± 17.4	19.6 ± 17.3	No significant change compared to placebo		
						CGI	3.9 ± 1.2	3.0 ± 1.4	No significant change compared to placebo		
						HAMD	10.5 ± 6.00	8.88 ± 6.79	No significant change compared to placebo		
Rybakowski et al.^62^	*n*_total_ = 8 (relatives of schizophrenic patients), *n*_STPD_ = 3	Open study	*n*_total_: first- or second-degree relatives of schizophrenia patients; *n*_STPD:_ diagnosis of STPD by DSM-IV criteria, impairment in social and occupational functioning >1 year	Manifested psychotic symptoms, previous pharmacologic treatment	Risperidone (0.5–2 mg/day)					The continuous treatment with a low-dose of risperidone administered for 2 to 3 years resulted in a significant improvement in social and occupational functioning and, in some of the subjects, in spectacular educational and professional achievements. In the subgroup of STPD patients, no side-effects besides transient sleepiness were reported. In 7 subjects, neuropsychological tests were obtained after 1 year of risperidone treatment. In all of them, a highly significant improvement compared with pretreatment period was observed on part B of Trail Making Test and part B of Stroop test as well as on all the subtests of Wisconsin Card Sorting Test	No concealment. No randomization. Various outcomes. Monocentric study. LoE: 3
Keshavan et al.^60^	*n*_STPD_ = 11	26-week open-label study	Diagnosis of STPD based on Structured Clinical Interview for DSM-IV (SCID)	Diagnosis of schizophrenia or schizoaffective disorder based on SCID, previous neuroleptic treatment for STPD, significant medical or neurological illness, mental retardation, and pregnancy or lactation	Low-dose olanzapine (average 9.32 mg/day)	HRSD24 total	19.7 ± 6.9	11.9 ± 6.4	7.8 ± 8.4^c^	Patients showed significant improvements in psychosis and depression ratings, as well as in overall functioning. Olanzapine was well tolerated, though significant weight gain was observed. Conclusion: This study provides preliminary data regarding olanzapine efficacy and tolerability in schizotypal personality disorder subjects. These data need to be confirmed in larger controlled clinical trials.^c^ The change in symptoms is measured as paired difference between baseline and posttreatment score	No concealment. No randomization. Various outcomes. Monocentric study. LoE: 3
						GAS	45.6 ± 11.5	59.6 ± 13.4	14.0 ± 9.0^c^		
						OAS	7.4 ± 5.6	2.1 ± 3.9	5.2 ± 6.0^c^		
						EPSE	0.65 ± 1.2	1.4 ± 2.2	0.75 ± 1.8		
						BPRS 18 total	44.8 ± 7.4	30.3 ± 7.8	−14.5 ± 7.5^c^		
Di Lorenzo et al.^63^	*n*_total_ = 43 (17 inpatients, 26 outpatients), *n*_STPD_ = 1 (inpatient)	Retrospective study	Diagnosis of schizophrenia-spectrum disorder		Aripiprazole	BPRS (for *n*_outpatients_)	60.62 ± 4.73	44.81 ± 4.53	Improvement of 54%	The final scores of the 2 scales showed a statistically significant difference from baseline (BPRS: *p* < 0.001 in the 2 groups; GAF: p < 0.005 in inpatients, *p* = 0.001 in outpatients). Inpatients and outpatients could improve in BPRS and GAF. Specific effects for patients with STPD are not mentioned	Retrospective study. No concealment. No randomization. Various outcomes. Monocentric study. LoE: 3
						BPRS (for *n*_inpatients_)	82.88 ± 5.13	56.65 ± 7.03	Improvement of 71%		
						GAF (for *n*_outpatients_)	37.65 ± 2.70	51.27 ± 3.15	Improvement of 35%		
						GAF (for *n*_inpatients_)	30.29 ± 3.80	50.06 ± 5.69	Improvement of 71%		
McClure et al.^53^	*n*_STPD_ = 31 (respectively 20 post drop-out)	2-week single-blind lead-in, then 10 week double-blind, randomized, placebo-controlled trial	STPD diagnosis by Diagnostic and Statistical Manual of Mental Disorders-Fourth Edition criteria	The exclusion criteria are not specifically named	Low-dose risperidone (0.25–2 mg/day)	PANSS general	26.9 ± 7.7	22.1 ± 5.2		There were no significant differences between the risperidone group and the placebo group in change from baseline in the PANSS or on any of the cognitive variables following either 6 weeks, all *F*s <0.5, all *p*s >0.15, or 12 weeks, all *F*s <1.2, all *p*s >0.28, of treatment	Various outcomes. Monocentric study. LoE: 2++
						PANSS positive	11.9 ± 4.7	9.4 ± 2.5			
						PANSS negative	13.9 ± 4.7	12.1 ± 5.2			
						WMS-VR	33.1 ± 5.1	34.9 ± 7.1			
						WMS-VR LD	29.1 ± 8.4	36.8 ± 13.5			
						WLL trail 5	12.8 ± 5.3	15.7 ± 5.3			
						WLL LD	10.9 ± 4.2	15.7 ± 6.6			
						CPT d′	0.9 ± 0.5	1.3 ± 0.83			
						PASAT	31.0 ± 5.9	36.7 ± 13.5			
						DOT 30 s delay	1.2 ± 1.4	0.91 ± 0.61			
						O-LIFE (for *n*_STPD_)	61.67 (9.41)				
Rabella et al.^54^	*n*_STPD_ = 9	Double-blind, randomized, placebo-controlled cross-over trial	Diagnosis of STPD using the Spanish version of the Structured Clinical Interview for the DSM-IV Axis II Personality Disorders (SCID-II)	Hospitalization or prior antipsychotic treatment	Risperidone (1 mg/day)	SPQ score	44.67 (6.75)			After placebo, STPD individuals showed slower reaction times to hits, longer correction times following errors and reduced ERN and Pe amplitudes. While risperidone impaired performance and decreased ERN and Pe in the control group, it led to behavioral improvements and ERN amplitude increases in the STPD individuals.^d^ The symptom change is measured as treatment × group interaction as Cohen’s *f*	Monocentic study. LoE: 2++
						O-LIFE	61.67 (9.41)				
						Eriksen Flanker Task: total responses		1015 ± 197	*F* = 1.42^d^		
						Eriksen Flanker Task: RT corrected errors		313 ± 88	*F* = 6.91^d^		
(B) Antidepressants											
Markovitz et al.^61^	*n*_total_ = 22, *n*_STPDonly_ = 4, *n*_STPD+BPD_ = 10	12-week, prospective, open-label trial	Diagnosis of BPD and/or STPD by DSM-III-R criteria	Psychopharmacological treatment other than lorazepam and chloral hydrate	Fluoxetine (20–80 mg/day)	HSCL score (for *n*_total_)	197.3 ± 60.1	69.5 ± 44.7	65% decrease	Treatment with fluoxetine leads to significant reduction in self-injury and Hopkins Symptom Checklist regardless of diagnosis	No concealment. No randomization. Various outcomes. Monocentric study. LoE: 3
						HSCL score (for *n*_STPD_)	159.8 ± 39.6	52.5 ± 30.4			
						HSCL score (for *n*_STPDandBPD_)	206.8 ± 54.4	68.5 ± 29.4			
Baer et al.^46^	*n*_total_ = 55 patients with OCD, *n*_STPD_ = 5	10-week double-blind placebo-controlled trial	YBOCS score ≥16; NIHM Global Scale score ≥7; HAMD score ≤16	Relevant Axis I or II disorders that may interfere with protocol compliance	Clomipramine hydrochloride 100–300 mg/day	YBOCS (for *n*_total_)	25.6 ± 6.7			No significant difference was noted on either YBOCS (*F* = 1.1, df = 1.53, *p* = 0.30) or NIHM Global Scale (*F* = 0.7, df = 1.53, *p* = 0.41) between patients with or without PD diagnoses at baseline. For Cluster A diagnoses, there was a significant YBOCS main effect (*F* = 6.2, df = 1.48, *p* = 0.02) after the intervention. STPD showed significant partial correlation with YBOCS (*r* = 0.31, *p* < 0.02) and significant Pearson's correlations with PRAS (*r* = 0.28, *p* < 0.05)	No randomization. Various outcomes. LoE: 2−
						YBOCS (for *n*_STPD_)	32.4 ± 2.9	27.8 ± 7.6			
						NIHM (for *n*_total_)	9.7 ± 1.9				
						HAMD (for *n*_total_)	5.4 ± 3.8				

^a^The descriptions of outcome are direct citations or extracts from the referred publications

^b^The symptom change is measured as standardized change: (pretreatment score−posttreatmentscore)/pretreatment standard deviation

^c^The change in symptoms is measured as paired difference between baseline and posttreatment score

^d^The symptom change is measured as treatment × group interaction as Cohen’s f

#### Individual outcomes

Most of the studies on an antipsychotic drug intervention found a positive effect in STPD (see Table 2A). Thiothixine reduced general symptoms (especially illusions, ideas of reference, paranoid ideation, cognitive deficits, and anxiety) in cohorts of mixed BPD and STPD patients, but the respective studies found no significant relationship between diagnosis and outcome of treatment.^47,57^ Risperidone was the only drug that was evaluated in studies that fulfilled relevant study quality criteria, such as reporting effect sizes, being randomized, and using quantitative measures and standardized questionnaires. It was evaluated in four independent studies: Koenigsberg et al. showed a reduction in the PANSS score in STPD but no difference in the STPD-specific SPQ;^50^ Rabella et al. reported an improvement in reaction time after treatment with risperidone;^54^ McClure et al. found no beneficial effect of risperidone in either the PANSS or cognitive measurements;^53^ and the open-label study by Rybakowski et al. found an increase in social and occupational functioning and cognitive tests after risperidone treatment.^62^ In an open-label augmentation trial, a small cohort (*n* = 11) of patients with STPD had significant improvements in psychosis and depression ratings when treated with olanzapine.^60^ In an open-label study in patients with comorbid OCD and STPD, a combination of fluvoxamine and olanzapine showed beneficial effects, i.e., it reduced the YBOCS score,^59^ and the concomitant diagnosis of STPD was significantly associated with a positive response. In a retrospective study, Di Lorenzo et al. reported a decrease of general symptoms when patients with schizophrenia spectrum disorder were treated with aripiprazole; patients with STPD were included, but no specific results were mentioned.^63^ In one case report, clozapine treatment reduced symptoms in a patient with comorbid OCD and STPD.^66^ Three studies investigating the use of antipsychotics analyzed mixed cohorts of STPD and BPD patients,^47,48,57^ and one study analyzed patients with schizophrenia spectrum disorder.^63^

Studies evaluating antidepressant treatment examined only mixed (BPD and STPD) or comorbid (OCD and STPD) cohorts (see Table 2B). In an open-label trial of fluoxetine, Markovitz et al. measured a reduction of general symptoms in patients with BPD, STPD, or both,^61^ regardless of the diagnosis. In another study, clomipramine showed no significant effects on OCD symptoms in a cohort of patients with OCD and STPD, and the authors reported a worse treatment outcome in patients with comorbid STPD than in those with other PD diagnoses.^46^ One case report described a patient with STPD who developed psychotic symptoms after treatment with fluoxetine,^64^ and other case reports showed positive effects of clomipramine, paroxetine, and buspirone on depressive or OCD symptoms^46,65^ (see Supplementary Tables 3C and D). Five studies tested other substances and their effect on cognitive impairments in patients with STPD (see Supplementary Table 3): On the basis of studies showing positive results of catecholamine agonistic treatment in schizophrenia-related disorders, Siegel et al. tested d-amphetamine in a cohort of patients with STPD and found an improvement in the Wisconsin Card Sorting Test (WCST); however, the authors found no effect of symptom change on the PANSS score.^56^ Two studies tested dopamine agonists (pergolide and dihydrexidine) and showed improvements in working and verbal memory, executive functioning, information processing, and divided attention.^52,55^ Two studies tested modulators of the autonomous nervous system (intravenous physostigmine, a cholinesterase inhibitor and enhancer of the parasympathetic nervous system, and guanfacine, an α_2A_ receptor agonist and sympatholytic drug) and their effect on cognition. These studies showed improvements in verbal memory, visuospatial working memory, and context processing.^49,51^ The sizes of the studies varied between 1 patient with STPD in a larger mixed cohort^63^ and up to 31 patients with STPD^53^ (see Supplementary Table 2).

### Articles on psychotherapy

#### Study characteristics

We identified one randomized clinical trial that compared integrated therapy with standard treatment,^68^ one uncontrolled clinical trial,^69^ and one case report of a patient with OCD and comorbid STPD.^70^ The sizes of the studies varied from 1^70^ to 79^68^ patients with a diagnosis of STPD (see Supplementary Table 2).

#### Individual outcomes

An uncontrolled prospective trial of a psychodynamic day-care treatment program found no benefit for STPD patients.^69^ One case report showed a reduction of symptoms in a patient with STPD and OCD^70^ after social skills training. Another trial also reported a positive effect of social skills training, i.e., STPD patients with this training had a lower transition rate from STPD to a psychotic disorder.^68^ We found no trial that evaluated cognitive behavioral therapy in STPD (see Table 3).

**Table 3:** Qualitative synthesis of psychotherapy

Reference	Study population	Study type	Inclusion criteria	Exclusion criteria	Intervention	Outcome^a^	Level of evidence (risks of bias LoE according to SIGN)
Karterud et al.^69^	*n*_total_ = 97, *n*_PD_ = 50, *n*_STPD_ = 13	Non-blinded, uncontrolled prospective trial	Diagnosis of Axis I or II psychiatric disorder, for *n*_STPD_: diagnosis according to DSM-III-R criteria	Acute psychosis, intense psychic suffering with long-standing incapacity to function in social or family roles	6-month psychodynamic day treatment including group psychotherapy (3× per week), art therapy groups (2× per week), body awareness group (2× per week), individual psychotherapy (1× per week), occupational therapy (1× per week)	The improvement of patients was measured by the change in symptoms (measured by General Symptom Index) and the Global Functioning (measured by Health Sickness Rating Scale). Whereas patients without personality disorder (change in GSI: 0.77 ± 0.50, change in HSRS: 8.9 ± 6.9, *p* < 0.01) improved the most, followed by the BPD and OCD groups, the improvement for patients with STPD was not statistically significant (change in GSI: 0.40 ± 0.77, change in HSRS: 0.2 ± 7.1). No patients committed suicide. One STPD patient made a suicidal attempt and was temporarily transferred to the acute ward. One STPD patient was transferred to long-term psychiatric hospital treatment	No concealment. No randomization. Various outcomes. Monocentric study. LoE: 3
McKay et al.^70^	*n*_STPD_ = 1 with comorbid OCD	Case report	Diagnosis of STPD by DSM-IV assessed by SCID-II; diagnosis of OCD by DSM-III-R criteria	No criteria	Social skills training plus exposure with response prevention (ERP)	The patient showed a decrease in OCD symptomatology as assessed by the YBOCS, as well as decreases in depression and anxiety as assessed by the Hamilton Rating Scale for Depression and the Hamilton Rating Scale for Anxiety. This is consistent with the hypothesis that OCD, when presented with comorbid schizotypal personality, is amenable to social skills interventions. At 6-month follow-up, the patient continued to have considerable symptoms, although his level of functioning had improved and remained at posttreatment levels	Case report. LoE: 3
Nordentoft et al.^68^	*n*_STPD_ = 79	Randomized clinical trial comparing integrated treatment with standard treatment	Diagnosis of STPD by the research criteria by the WHO 1993 (ICD-10)	Overt psychotic symptoms	Integrative vs. standard treatment	In the multivariate model, male gender increased risk for transition from STPD to psychotic disorder (relative risk = 4.47, (confidence interval 1.30–15.33)), while integrated treatment reduced the risk (relative risk = 0.36 (confidence interval 0.16–0.85)). Significantly more patients in integrated treatment than in standard treatment were treated with antipsychotic medication. Integrative treatment included assertive community treatment, social skills training, family involvement, and psycho-education	No concealment. Monocentric study. LoE: 2

^a^The descriptions of outcome are direct citations or extracts from the referred publications

### Articles on longitudinal course and follow-up studies

#### Study characteristics

We included eight studies of clinical cohorts.^71–78^ Studies with mixed clinical and non-clinical cohorts were included if they predicted the development of an STPD diagnosis from childhood to adulthood or the conversion rate to psychotic illness.^79–81^ One article focused of the stability of a diagnosis of STPD in young adult twins.^20^ The study size depended on the investigated cohort (for details see Supplementary Table 2).

#### Individual outcomes

The follow-up periods of the studies ranged from a minimum of 1 year^81^ to 27 years.^80^ In a mixed cohort of healthy and mentally ill children, Bernstein et al. showed that an early STPD diagnosis during adolescence rarely persisted over a 2-year follow-up period.^79^ Along the same lines, in their study in adults Grilo et al. reported a remission of the STPD diagnosis in 61% of patients and suggested that, although maladaptive traits may persist, the severity of symptoms can change.^76^ In contrast, Asarnow et al. showed that children with a diagnosis of STPD mostly kept the diagnosis, but 25% of them developed more severe schizophrenia spectrum disorders (schizophrenia or schizoaffective disorder).^81^ Olin et al. evaluated the relationship between personality traits and disorders in a community-based cohort of healthy and mentally ill children and concluded that early traits can be predictive for the development of an STPD. Four studies evaluated longitudinal global functioning and impairment:^71–74^ Impairments in global functioning and social impairment differed depending on the study sample, but, overall, patients with pure STPD had less impairment than patients with other comorbid PDs or SZ. In particular, patients with comorbid BPD had poor global functioning. Treatment with antipsychotic medication was common in STPD patients.^74,75^ They were also frequently hospitalized and received psychotherapy.^75^ Two studies evaluated the stability of symptoms over time: McGlashan et al. found paranoid ideation and unusual experiences to be the most stable, whereas oddness appeared to be the least prevalent and most changeable;^78^ and Kendler et al. studied the stability of Cluster A PDs in twins and concluded that genetic risk factors lead to highly stable subtypes of the disorder, whereas shared environmental risk factors lead to rather transient symptoms^20^ (see Table 4). Two studies focused on the conversion from STPD to a psychotic illness and found rates of 25–48%.^24,82^

**Table 4:** Qualitative synthesis of longitudinal outcome studies of patients with schizotypal personality disorder

Reference	Study population	Follow-up period	Outcome^a^
Plakun et al.^71^	*n*_total_ = 237 psychiatric patients, *n*_STPD_ = 13, *n*_STPD+BPD_ = 6	14 years	Diagnoses in this study were based on DSM-III criteria. Patients suffering from STPD without comorbid major affective disorder functioned better than patients with SZ. They had higher scores in global functioning (GAS) than SZ patients at baseline but not at follow-up. STPD patients with comorbid BPD were as impaired as schizophrenics at admission but significantly better at follow-up
McGlashan^72^	*n*_total_ = 253 psychiatric patients, *n*_STPD_ = 10, *n*_STPD+SZ_ = 61, *n*_STPD+SZ+BPD_ = 30, *n*_STPD+BPD_ = 18	Range: 2–32 years	STPD as defined by DSM-III criteria appeared to be common in the Chestnut Lodge follow-up study patients, although it was rare as a pure syndrome. From the perspective of follow-up, STPD seemed to be related to SZ but not to BPD. The mixed Axis II borderline syndrome (STPD+BPD) had a long-term profile closer to BPD than to STPD. The cohorts meeting STPD criteria had relatively poor social adjustments and fewer social contacts. The pure STPD cohort achieved the highest level on education compared to the mixed diagnoses. The pure STPD sample was mainly single (70%) and male (60%). Premorbid functioning was poor socially and good instrumentally
Modestin et al.^73^	*n*_total_ = 39 psychiatric patients, *n*_STPD_ = 14 (7 for follow-up), *n*_SZ_ = 25 (17 for follow-up)	4 years	Diagnosis of STPD was based on DSM-III, of SZ on ICD-9 and parts on DSM-III. A relationship not only between STPD and SZ but also between STPD and BPD could be detected. Pure STPD patients are rarely dysfunctional and less likely to require hospital care. Therefore, the clinical sample investigated is small and might not be representative for all STPD patients. On a blind examination, STPD patients in this cohort were found to be less socially adjusted and they tended to be more symptomatic. Compared with a small DSM-III schizophrenia subgroup, STPD patients undertook more suicide attempts. STPD patients were rating higher in social dissatisfaction. Patients with STPD were more anxious and they tended to suffer more from obsessive-compulsive symptoms and depression. Transient psychoses were frequent in STPD patients. The average neuroleptics dose was twice as low in STPD compared to SZ (92% of *n*_total_ received neuroleptic medication)
Mehlum et al.^74^	*n*_total_ = 97 patients with PD, *n*_STPD_ = 13 at admission, *n*_STPD_ = 9 at discharge	range: 1.6–4.9 years	STPD diagnoses were made according to DSM-III-R at index hospitalization and by SCID interview at follow-up. STPD patients displayed a moderate symptom reduction after 3 years of treatment but retained relatively poor global functioning. They were least socially adjusted, employed, and self-supporting of all diagnostic subgroups. STPD and BPD patients had far more inpatient treatment than other PDs
Bernstein et al.^79^	*n*_total_ = 733 community-based adolescents	2 years	The overall prevalence of personality disorders peaked at age 12 years in boys and at age 13 years in girls and declined thereafter. STPD was the least prevalent Axis II disorder (moderate STPD 1.8%, severe STPD 1.2%). Children who met the criteria for STPD had increased social impairments, school or work problems, and a higher comorbidity with Axis I disorders. Longitudinal follow-up revealed that most Axis II disorders did not persist over a 2-year period. Subjects with disorders identified earlier remained at elevated risk for receiving a diagnosis again at follow-up (persistence after 2 years: for moderate STPD 9%, for severe STPD 11%)
Olin et al.^80^	*n*_total_ = 232 children, *n*_STPD_ = 36 children	Range: 15–27 years, based on teachers’ school reports	The lifetime diagnoses used in the study are based on DSM-III-R. The first assessment was at age 15 years, the second at age 25 years, and the third between age 39 and 42 years. Those who later developed STPD were found to be more passive and unengaged and more hypersensitive to criticisms compared with the non-schizophrenia groups according to school reports. Males who developed STPD were found to be less disruptive and hyper-excitable compared with males with schizophrenia; females with STPD did not differ from females with schizophrenia. A receiver operating characteristic analysis found these factors to predict 73.5% of future STPDs. The three major factors accounting for 54.4% of the variance were labeled as “socially anxious and withdrawn,” “disruptive and hyper-excitable,” and “passive and unengaged.” These findings suggest that pre-schizotypal traits may be identified in late childhood or adolescence
	Comparative groups: *n*_comp1_ = 31 SZ children, *n*_comp2_ = 37 nonpsychotic but mentally ill children, *n*_comp3_ = 68 children not mentally ill but schizophrenic mother, *n*_comp4_ = 60 healthy children		
Grilo et al.^76^	*n*_total_ = 633 patients, *n*_PD_ = 544, *n*_MDD_ = 89, *n*_STPD_ = 78	2 years	The study examined the stability of different personality disorders over time. The STPD remission rate was 61% after 24 months. Remission rates after a more stringent definition with two or fewer criteria (by the DSM–IV Personality Disorders Follow Along Version, DIPD-FAV) after 12 consecutive months was 23% for STPD. Dimensionally, these findings suggest that PDs may be characterized by maladaptive trait constellations that are stable in their structure (individual differences) but can change in severity or expression over time
Warner et al.^77^	*n*_total_ = 376 patients with PD	2 years	This study explores the extent to which relevant personality traits are stable in individuals diagnosed with four personality disorders (schizotypal, borderline, avoidant, and obsessive-compulsive personality disorders). The PDQ-IV was used for screening. The DIPD-IV was used for making the initial diagnosis based on DSM-IV criteria. The test–retest kappa for STPD was 0.64. There was an insufficient sample size in the inter-rater reliability sample to calculate the kappa for STPD, but diagnostic agreement was 100%. Participants were interviewed at 6 months, 1 year, and 2 years following the baseline assessment. Changes in personality traits were determined via a re-administration of the NEO–PI–R at the 1- and 2-year follow-up. The DIPD–IV was modified to record the presence of each criterion for the four PDs for each month of the follow-up interval. The standardized parameter estimates reflecting the stability for the latent trait variable across time were significant and quite large (*β*= 0.76 and *β* = 0.83, both *p*s < 0.01) as were the stability estimates for STPD (*β* = 0.90 and *β* = 0.81, both *p*s < 0.01). The results demonstrate significant cross-lagged relationships between trait change and later disorder change for three of the four personality disorders studied
Asarnow et al.^81^	*n*_STPD_ = 12 children, *n*_SZ_ = 18 children	range: 1–7 years	There was significant continuity between SZ spectrum disorders in childhood and adolescence. The most common clinical outcome for children with STPD was continuing STPD, supporting the hypothesis of continuity between childhood and later STPD. However, 25% of the STPD sample developed more severe SZ spectrum disorders (schizophrenia or schizoaffective disorder, also supporting the hypothesis that STPD represents a risk or precursor state for more severe SZ spectrum disorders
McGlashan et al.^78^	*n*_total_ = 474 patients with personality disorders, *n*_STPD_ = 85	2 years	In this study, a 24-month follow-up was obtained to evaluate the change of personality disorder criteria over time. For STPD, the most prevalent and least changeable criteria over 2 years were paranoid ideation, and unusual experiences. The least prevalent and most changeable criteria were odd behavior and constricted affect
Woods et al.^82^	*n* = 377 patients with prodromal syndrome, *n*_HSC_ = 196, *n*_FHR_ = 40, *N*_STPD = _49	2.5 years	40% of prodromal patients converted to fully psychotic illness during 2.5 years of follow-up. Corresponding rates for help-seeking comparison (HSC) group, familial high-risk (FHR) group, and STPD subjects were correspondingly 4, 0, and 36%. Cox regression comparing distinguished prodromal patients from HSC but not from STPD subjects
Debbane et al.^24^	*n* = 376 patients by a clinically relevant expression of schizotypy (i.e., STPD, schizoid PD, or SD	range: 2–20 years	The conversion rates from STPD to a psychotic disorder varied between 25% and 48%. Suspected STPD in children, however, seldom led to the later emergence of a schizophrenic-spectrum psychotic disorder (only 6.25%)
Kendler et al.^20^	*n*_total_ = 2282 twins	range: 6–11 years	The study examines the stability of genetic and environmental factors in paranoid and schizotypal PD. The stability over time of the criteria counts for STPD, estimated as polychoric correlations, was +0.40. 71% of the temporal stability derived from the effect of genetic factors. Shared genetic risk factors for two of the Cluster A PDs are highly stable in adults over a 10-year period while environmental risk factors are relatively transient. Over two thirds of the long-term stability of the common Cluster A PD liability can be attributed to genetic influences

^a^The descriptions of outcome are direct citations or extracts from the referred publications

### Quality of the studies

The composition and size of the cohorts varied greatly across the studies, and all treatment studies were single center. Some studies in mixed samples of patients reported results on very few or only single STPD patients. It remains questionable whether the results of these studies can be applied to a larger group of patients with STPD. Most of the articles did not mention disease severity because of a lack of a suitable measurement tool. A variety of methods for evaluating the quality of diagnostic instruments, the outcome of clinical interventional studies (drug treatment or psychotherapy), or longitudinal outcome were applied across the studies, resulting in a high heterogeneity and hampering comparability.

## Discussion

We present a systematic review of diagnostic instruments and pharmacological and psychosocial treatment strategies for STPD. After performing a standardized and systematic literature search and analysis, we assessed 94 full-text articles for eligibility and evaluated 54 of them. At this point, it would be premature to make clear recommendations for the use of specific diagnostic instruments or drug or psychotherapy treatment approaches. Our evaluation of diagnostic instruments made clear that the diagnosis of STPD has changed over time. In each diagnostic system, the threshold for meeting symptom criteria seems to differentiate between related diseases, such as other PDs or schizophrenia. Nearly all the diagnostic instruments discussed for STPD have adequate inter-rater and test–retest reliability. Our review confirms that the SIDP for DSM-III, SIDP-R for DSM-III-R, and SCID-II for DSM-IV are suitable for diagnosing STPD, but we found that the diagnostic tool PDQ-4+ is more suitable for screening. Factor analysis models are frequently discussed as a diagnostic alternative to catalogs with rather arbitrary diagnostic criteria. A complete review of the scales used to access schizotypy as a broad concept can be found in the review by Mason, which focuses not only on the clinical diagnosis but also on psychometric measurements that assess schizotypal personality traits and define a high-risk group for schizophrenia.^5^ Longitudinal studies examined the stability of the diagnostic entity and observed moderate-to-high remission rates between an early childhood onset and later adulthood. Patients suffering of comorbid BPD showed poorer social functioning than patients with pure STPD.^71,72^ Yet, drug treatment responses in patients with comorbid BPD were regardless of the diagnosis.^47,57,61^ Patients suffering of a comorbid OCD showed better response rates after treatment with olanzapine^59^ and poorer responses after clomipramine treatment.^46^ The articles on treatment clearly showed that antipsychotics are the most frequently used drugs. When we considered only studies that were of acceptable methodological quality (see LoEs in Tables 2 and 3 and Supplementary Table 3 and 4), risperidone had the best, but still limited, evidence for reducing clinical symptoms in patients with STPD. Antidepressants have only been tested in mixed cohorts (patients with comorbid OCD or BPD), making it difficult to draw definite conclusions on their effectiveness in STPD. Most of the drug treatment studies were conducted in patients with STPD and a comorbid disorder, which also limits our ability to draw conclusions. Moreover, some studies included only a few patients, resulting in an LoE of 2− to 3. Remarkably, to the best of our knowledge no treatment guidelines or Cochrane Collaboration reviews exist that provide a comprehensive discussion of treatment alternatives for STPD. The WFSBP Guidelines for Biological Treatment of Personality Disorders provide only general recommendations for antipsychotics on the basis of minimal evidence from clinical trials and expert opinion (Herpertz et al. 2007).

In summary, our systematic review shows that the best evidence for efficacy in STPD is available for risperidone and to a limited extent for olanzapine. The literature on psychotherapy is sparse and does not allow us to make any recommendations, although social skills training seems to be effective and should be offered to patients with STPD. Large-scale naturalistic and interventional trials with defined diagnostic cohorts and strict study designs are needed to provide the data for more detailed evidence-based recommendations.

## Methods

### Study selection

This systematic review was conducted by searching the PubMed/MEDLINE databases for papers published at any time. We conducted the final search on September 14, 2016, at which time the data source contained studies from April 1, 1947, to August 21, 2016. A total of 145 combinations of search terms were used to search the databases with the ENDNOTE X7 search tools (see Supplementary Information). Duplicates were removed by using the ENDNOTE X7 duplication detection feature. The publications (titles and abstracts) were then screened for relevance. To be included, the articles had to report on studies of original data and focus on the diagnosis, treatment, or follow-up of patients with STPD. Because including only studies with a Scottish Intercollegiate Guidelines Network (SIGN) LoE of 1− to 1++ would have limited the number of studies available for inclusion, we included not only randomized control trials but also cohort studies, retrospective non-analytical studies, and case studies (LoE 2++ to 3). Expert opinions (LoE 4) were not considered. Study designs and LoE grading are described in the Tables 2A, B, and 3, and Supplementary Tables 3 and 4. Reviews, meta-analyses, and non-English publications were excluded. Two reviewers independently analyzed the full-text publications and retrieved data on clinical diagnosis and treatment. We searched also three additional databases (WHO Clinical Trials (http://apps.who.int/trialsearch/), ClinicalTrials (https://clinicaltrials.gov/), and the Cochrane Library (http://www.cochranelibrary.com/)) for ongoing or planned clinical trials and for systematic reviews or meta-analyses. Using the search term “schizotypal personality disorder,” we identified 18 clinical trials. Furthermore, we found two additional trials that had been completed; however, upon closer inspection it became clear that they did not meet the inclusion criteria for this systematic review. We identified one protocol^83^ in the Cochrane Library, but it was withdrawn with no results in 2014.

Our search strategy yielded 3420 unique studies, 3326 of which were excluded after we had screened the titles, abstracts, and article format reviews, resulting in 94 full-text articles. These 94 articles were scanned for the inclusion criteria of this systematic review—to be included, studies had to investigate diagnostic instruments for or the treatment or longitudinal course of STPD. After full-text screening, 38 articles had to be excluded because they did not include original data or were not about clinical patients with STPD. The remaining 56 articles were sorted into the following categories: clinical diagnostic instruments (18 studies), pharmacological treatment (22 studies), psychotherapy (3 studies), and longitudinal course and follow-up (13 studies) (see Supplementary Information). Publications on diagnostic questionnaires were only included if they evaluated diagnostic criteria or questionnaires as assessment tools. Articles on factor analysis models alone were excluded, and publications on drug treatment were excluded if there was no report of a clinical outcome. We identified three overlapping patient cohorts: one in the articles on diagnostic instruments (Battaglia et al.^35^ and Fossati et al.^44^); one in the articles on drug treatment (Goldberg et al. 1986^47^ and 1987^48^); and one in the Chestnut Lodge cohort (McGlashan et al. published an article on testing DSM-III criteria^29^ and a 2-year follow-up study in a separate article^72^).

### General study characteristics

We assumed a large heterogeneity in disease severity among the included patients who were recruited in outpatient and inpatient settings. Because of the inconsistent outcome measures in the interventional groups, in our view the available data were not suited to perform quantitative analyses, e.g., with a meta-analytical approach. Some study populations included also healthy and population-based individuals, but these studies were only taken into consideration when a clinical diagnosis of STPD was mentioned.^20,34,36,38,79,80^ Most of the studies focused on adults, although three focused on children and the longitudinal course of their diseases.^79–81^ The study sizes varied greatly, as indicated in Supplementary Table 2.

## Electronic supplementary material

Supplementary Information

## Data Availability

This is a systematic review. All data generated or analyzed during this study is included in this published article (or Supplementary Information). No other data are available.

## Electronic supplementary material

**Supplementary information** accompanies the paper on the *npj Schizophrenia* website (10.1038/s41537-018-0062-8).
