# Supplementary material for: Diagnosis and treatment of schizotypal personality disorder: evidence from a systematic review
Source: NPJ Schizophr. 2018 Oct 3;4:20. doi: 10.1038/s41537-018-0062-8 (PMC6170383; doi:10.1038/s41537-018-0062-8)
Supplement: Supplementary file 1 — Supplementary Information [file 41537_2018_62_MOESM1_ESM.doc]

**22596** records identified through systematic database search

**3420** records after duplicates removed

**3326** articles were excluded on title, abstract, and format review

(reviews / non-english)

**3420** records screened on title and abstract

**94** full-text articles assessed for eligibility

**38** articles were excluded on full-text

(review / meta-analysis on other PDs /no clinical patients or clinical outcomes)

**56** articles included in qualitative synthesis:

- **18** studies on clinical diagnosis

- **22** studies on drug treatment

- **3** studies on psychotherapy

- **13** studies on follow-up

**Figure 1:** PRISMA diagram summarizing the flow of information through all phases of this systematic review

**Table 1 Search term Hits in Pubmed (last search at September 9^th^ 2016))**

|  | schizotypy | schizotypal | STPD | | schizotypal AND  personality AND disorder | schizotypal PD |
| --- | --- | --- | --- | --- | --- | --- |
|  |  |  |  |  | |  |
| diagnos* | 510 | 2145 | 15 | 1993 | | 213 |
| study | 605 | 1732 | 73 | 1513 | | 170 |
| clinical trial | 37 | 145 | 6 | 132 | | 46 |
| DSM-IV | 40 | 305 | 7 | 279 | | 69 |
| DSM-V | 27 | 191 | 5 | 175 | | 43 |
| ICD-10 | 3 | 78 | 0 | 51 | | 7 |
| comorbidity | 30 | 229 | 1 | 213 | | 58 |
| symptom* | 361 | 1084 | 3 | 939 | | 126 |
| treatment | 106 | 903 | 34 | 813 | | 155 |
| intervention | 12 | 120 | 2 | 110 | | 27 |
| psychoactive drugs | 2 | 3 | 0 | 3 | | 2 |
| neuroactive drugs | 0 | 0 | 0 | 0 | | 0 |
| evidence-base* | 1 | 12 | 0 | 11 | | 2 |
| therap* | 12 | 239 | 0 | 218 | | 72 |
| antpsychotic | 16 | 193 | 0 | 176 | | 88 |
| antidepressant | 2 | 58 | 0 | 56 | | 21 |
| mood stabilizer | 0 | 2 | 0 | 2 | | 0 |
| SSRI | 2 | 35 | 0 | 32 | | 16 |
| SNRI | 0 | 0 | 0 | 0 | | 0 |
| medication | 21 | 112 | 4 | 94 | | 37 |
| compound* | 11 | 18 | 1 | 14 | | 4 |
| psychotherapy | 32 | 242 | 1 | 215 | | 21 |
| behavioral | 344 | 1088 | 9 | 979 | | 94 |
| psychoanalysis | 1 | 13 | 0 | 11 | | 0 |
| socio therapy | 1 | 4 | 0 | 3 | | 1 |
| neuro stimulation | 2 | 1 | 0 | 0 | | 1 |
| ECT | 0 | 2 | 0 | 2 | | 0 |
| TMS | 0 | 0 | 0 | 0 | | 0 |
| TDCS | 0 | 1 | 0 | 0 | | 0 |
| cogniti* | 418 | 814 | 1 | 715 | | 47 |
| **sum** | **2596** | **9769** | **162** | **8749** | | **1320** |

| **Table 2 Cohort sizes** | |  |  |  |
| --- | --- | --- | --- | --- |
|  |  |  |  |  |
| **Study Population** | **Diagnostic** | **Drug treatment** | **Psychotherapy** | **Follow-up** |
| **Community-based subjects** | |  |  |  |
| n_min_ | 195 |  |  | 232 |
| n_max_ | 4000 |  |  | 2282 |
| mean | 1499.00 |  |  | 906,00 |
| SD | 2166.59 |  |  | 815,08 |
| **Psychiatric Patients** | |  |  |  |
| n_min_ | 19 | 22 | 97 | 29 |
| n_max_ | 721 | 55 | 97 | 668 |
| mean | 298.81 | 42.14 | 97.00 | 279,44 |
| SD | 233.82 | 13.90 | 0.00 | 193,60 |
| **STPD patients** |  |  |  |  |
| n_min_ | 2 | 1 | 1 | 10 |
| n_max_ | 166 | 31 | 79 | 86 |
| mean | 40.67 | 10.10 | 31.00 | 34,44 |
| SD | 47.84 | 9.00 | 42.00 | 28,10 |

| **Table 3 Qualitative Synthesis of Drug Treatment Trials** | | | | | | |  |  |  |  |  |
| --- | --- | --- | --- | --- | --- | --- | --- | --- | --- | --- | --- |
|  | | |  |  |  |  |  |  |  |  |  |
| **Year and Reference** | **Study Population** | **Study Type** | **Inclusion Criteria** | **Exclusion criteria** | **Intervention** | **symptoms measured** | **mean pre treatment** | **mean post treatment** | **symptom change** | **Outcome^1^** | **Level of Evidence**  **(Risks of bias**  **LoE according to SIGN)** |
| 1994  Zwier et al. | n_STPD_ = 1 with comorbid social phobia and mixed personality disorder | case report | diagnosis of STPD by MMPI |  | buspirone (5 - 20 mg/d) | BPRS | 36 | 24 |  | The patient showed improvement in social phobia and resolution of mild psychotic symptoms. No significant side effects occurred. The patient also showed improvement of social functioning after one year of treatment. | Case report  **LoE: 3** |
|  |  |  |  |  |  | HAM-A | 5 | 0 |  |  |  |
| 1996  Siegel et al. | n_STPD_ = 9 | 9-day, double-blind, placebo-controlled trial | diagnosis of STPD by Schedule for Affective Disorders (Spitzer and Endicott, 1975) and with the Schedule for Interviewing DSM-III Personality Disorders (SIDP) | seizure disorder, substance dependence or serious substance abuse, abnormalities on physical examination or on laboratory tests, psychoactive medication for a minimum of 2 weeks prior to the first challenge | d-amphetamine (dopamine and norepine-phrine agonist) (30 mg) | PANSS positive | 12.4 ± 4.5 |  |  | Patients, particularly those who made more perseverative errors, demonstrated amphetamine-associated improvement on WCST performance. The data in this preliminary study suggest that some of the cognitive dysfunctions present in STPD may improve with amphetamine challenge. | Monocentric study  **LoE: 2++** |
|  |  |  |  |  |  | PANSS negative | 12.3 ± 5.3 |  |  |  |  |
|  |  |  |  |  |  | WCST perserverative errors: |  | 11.1 ± 8.4 | Improve-ment in task perfor-mance correlated with placebo perse-verative errors (r = 0.84, n = 9, p=0.005) |  |  |
| 2001  Kirrane et al. | n_STPD_ = 10 | single administration, double-blind placebo-controlled trial | diagnosis of STPD by DSM-III-R criteria using the Schedule for Affective Disorders and Schizophrenia (SADS) and the Structured Interview for DSM-III-R Personality (SIDP-R) | medical illness, past substance dependence, substance abuse in the previous six months, previous psychoactive medication in the last month | intravenous physostigmine 0.014 mg/kg:  a) short infusion for 20 min (n = 6)  b) long infusion for 60 min (n = 8) | Visuospatial working memory (Dot test) |  |  | short infusion:  0.63 ± 0 for 20 min | Physostigmine tended to improve the Dot test, but not serial verbal learning performance in these patients. Physostigmine infused over 20 min tended to improve working memory at the 20 and 30 s delay conditions of the Dot test compared with placebo (drug vs. placebo paired t-test at 20 s delay: t = 2.0, d.f. 5, p = 0.09; paired t-test for 30 s delay: t = 2.2, d.f. 5, p = 0.07). While physostigmine infused over  60 min had a non-significant effect over placebo (drug vs. placebo paired t-test: t < 1, d.f. 7, p = n.s. both delays). | Monocentric study  **LoE: 2++** |
|  |  |  |  |  |  |  |  |  | short infusion: 0.83 ± 1. for 30 min |  |  |
| 2007  McClure et al. | n_STPD_ = 29 | 4-week, randomized parallel-design, double-blind, placebo-controlled trial, followed by 4-week open-label extension | diagnosis of STPD by DSM-IV criteria | psychotic disorder or bipolar I disorder, substance dependence or abuse in the preceding 6 months, psychotropic medications. | guanfacine (a_2A_ agonist, up to 2mg/d) | Maintenance of Context with the AX-CPT: | | | | At the end of double-blind treatment, participants treated with guanfacine demonstrated a significant reduction in BX errors and a small but significant increase in AY errors, a pattern that was not seen in the participants treated with placebo. Conclusions: STPD participants improved in their context processing toward a normal response bias, making fewer BX and more AY errors, after being treated with guanfacine. | Monocentric study  **LoE: 2++** |
|  |  |  |  |  |  | BX errors | 14.1 ± 28.6 | 6.7 ± 12.8 |  |  |  |
|  |  |  |  |  |  | AY errors | 4.5 ± 7.6 | 6.5 ± 10.4 |  |  |  |
|  |  |  |  |  |  | other errors did not show significant difference between treatment and placebo | | | |  |  |
| 2010  McClure et al. | n_STPD_ = 25 | 4-week, double-blind, placebo-controlled trial | diagnosis of STPD by DSM-IV criteria | diagnosis of schizophrenia, any schizophrenia-related psychotic disorder or bipolar disorder, alcohol or substance abuse within the last 6 months or history of past substance dependence, psychotropic medication 2 weeks prior to the study | pergolide (dopamine agonist targeting D_1_ and D_2_ receptors, 0.025 -0.3 mg/d) | Verbal working memory measured by letter number span | 15.33 ± 3.2 |  | change score = 1.83 ± 2.25 | The pergolide group showed significant improvements in verbal working memory, verbal memory, and executive functioning. These results suggest that dopamine agonists may provide benefit for the cognitive abnormalities of schizophrenia spectrum disorders. Other cognitive tests, such as PASAT (for verbal working memory), WMS delayed recall (for visual-spatial long-term memory), WLL immediate recall (for verbal memory), AX-CPT BX short and long delay (Context processing), and TMT Part A (for processing speed) showed no significant changes between treatment and placebo group. | Various outcomes  Monocentric study  **LoE: 2+** |
|  |  |  |  |  |  | Verbal memory measured by WLL delayed recall | 16.57 ± 5.10 |  | change score = 3.00 ± 2.0 |  |  |
|  |  |  |  |  |  | Executive functioning measured by TMT Part B (s) | 83.42 ± 34.06 |  | change score = 31.75 ± 39.61 |  |  |
| 2015  Rosell et al. | n_STPD_ = 16 | 4-day, double-blind, placebo-controlled trial | diagnosis of STPD by DSM-IV criteria using the Structured Clinical Interview for DSM-IV Axis I and II Disorders | significant medical/neurological problems, pregnancy or nursing, diagnosis of major psychotic disorders (such as bipolar I or schizophrenia), or major depressive episode, active suicidal ideation, alcohol or illicit substance abuse within past six months, past history of substance dependence, cognitive impairment, as defined by scoring at or below the 25th percentile of the published scores for healthy participants on at least one test of working-memory battery that included the PASAT, N-back test, and Dot test | Dihydrexidine (DAR-0100A, D1 Dopamine Receptor Agonist) or 15 mg/150 ml or normal saline administered intravenously over 30 min | PASAT | 28.5 ± 12.2 | 34.6 ± 12.6 | change score (in %): 26.0 ± 14 | Treatment with DAR-0100A was associated with significantly improved PASAT performance relative to placebo, with a very large effect size (Cohen’s d = 1.14). Performance on the N-back ratio was also significantly improved; however, this effect rested on both a non-significant enhancement and diminution of 2-back and 0-back performance, respectively; therefore interpretation of this finding is more complicated. DAR-0100A was well tolerated. | Various outcomes  Monocentric study  **LoE: 2+** |
|  |  |  |  |  |  | n-back |  |  |  |  |  |
|  |  |  |  |  |  | 2-back : 0-back ratio | 0.84 ± 0.15 | 0.93 ± 0.11 | change score (in %): 12.1 ± 9 |  |  |
|  |  |  |  |  |  | 2-back | 0.78 ± 0.14 | 0.82 ± 0.13 | change score (in %): 5.66 ±8. |  |  |
|  |  |  |  |  |  | 0-back | 0.94 ± 0.06 | 0.89 ± 0.09 | change score (in %): - 5.54 ± 6.74 |  |  |
|  |  |  |  |  |  |  |  |  |  |  |  |
|  |  |  |  |  |  |  |  |  |  |  |  |
|  |  |  |  |  |  |  |  |  |  |  |  |
|  |  |  |  |  |  |  |  |  |  |  |  |

^1^ The descriptions of outcome are direct citations or extracts from the referred publications.

| **Table 4 Case Reports of Drug Treatment** | | | | | | | | | | | | | | |  | |
| --- | --- | --- | --- | --- | --- | --- | --- | --- | --- | --- | --- | --- | --- | --- | --- | --- |
|  | | | | | |  | | |  | | |  |  | |  | |
| **Year and Reference** | **Study Population** | **Study Type** | **Inclusion Criteria** | **Exclusion criteria** | **Intervention** | | **symptoms measured** | **mean pre treatment** | | **mean post treatment** | **Outcome^1^** | | | **Level of Evidence**  **(Risks of bias**  **LoE according to SIGN)** | |  |
| 2008  Poyurovsky et al. | n_STPD_ = 2 with comorbid OCD | case report |  |  | patient 1: clozapine (12.5 - 150 mg/d);  patient 2: clozapine (12.5 - 250 mg/d) | | YBOCS (patient 1)  YBOCS (patient 2) | 40  40 | | 15  26 | Relatively low-dose clozapine treatment can lead to reduction of clinical symptoms in patients with OCD and comorbid STPD. | | | Case report  **LoE: 3** | |  |
| 1998  Gutkovich  et al. | nSTPD = 1 | case-report |  |  | fluoxetine (20 mg/d) | |  |  | |  | After the treatment with fluoxetine the patient developed transient psychotic symptoms and polydipsia. | | | Case report  **LoE: 3** | |  |
| 1998  Heiden et al. | nSTPD = 1 (with concomitant OCD and anorexia nervosa) | case-report |  |  | paroxetine (60 mg per day) in combination with the antipsychotic haloperidol (3 mg per day) | |  |  | |  | Within 6 weeks, the symptoms of anorexia and her OCD behavior, measured using the Y-BOCS, had improved. Shortly after she was discharged, however, her medication was discontinued and her symptoms gradually worsened. The treatment was changed to paroxetine (60 mg per day) and pimozide (2-4 mg per day), which have been given for 9 months. Since then. she suffers from no psychopathological problems, apart from an insignificant subdepressive mood, a somewhat reduced drive and some minor compulsive symptoms. | | | Case report  **LoE: 3** | |  |

^1^ The descriptions of outcome are direct citations or extracts from the referred publications.
